# Effective of high-intensity interval training and moderate-intensity continuous training on body composition, glycolipid metabolism, and cardiopulmonary function in patients with pre-diabetes: a randomized controlled trial

**DOI:** 10.3389/fendo.2025.1614149

**Published:** 2025-07-29

**Authors:** Xinbei Chen, Lijuan Wu, Yan Zheng, Xuling Ni, Xiaojin Zhuang, Liming Chen, Qiaoling Hu, Chunyan Zou, Lianhua Yin

**Affiliations:** Health Management Center, The Second Affiliated Hospital of Fujian University of Traditional Chinese Medicine, Fuzhou, Fujian, China

**Keywords:** prediabetes, high-intensity interval training, body composition, glycolipid metabolism, cardiopulmonary function

## Abstract

**Aims:**

The aim of this study is to compare the effectiveness of high-intensity interval training (HIIT) and moderate-intensity continuous training (MICT) on body composition, cardiovascular function, glycolipid metabolism, and cardiopulmonary function in patients with pre-diabetes.

**Methods:**

Seventy-one participants were randomly assigned to the HIIT (10 × 1-min at 75%–90% HR_peak_, intersperse with 1-min active recovery at 50% HR_peak_) or MICT (50 min at 55%–70% HR_peak_) for a 12-week (three times per week) program. The outcome measured was the change in body composition, cardiovascular index, glycolipid metabolism, and cardiopulmonary. The trial was registered on the Clinical Trial Registry (ChiCTR1900026905).

**Results:**

The body mass index decreased in the HIIT (*P* = 0.016) and MICT (*P* = 0.021) groups. The participants in the MICT group had a significantly decreased in visceral adipose area (*P* = 0.043) and body fat rate (*P* = 0.030) after training, compared with the HIIT group. Analysis of systolic blood pressure revealed statistical difference in the HIIT and MICT interventions (*P* < 0.001), but there was not statistical difference between groups (*P* = 0.398). MICT was better than HIIT in reducing diastolic blood pressure (*P* = 0.011). The significant effect of fasting blood glucose, 2-h glucose, and glycated hemoglobin (HbA1c) showed an obvious descent in the HIIT and MICT groups (*P* < 0.001). Regarding the blood lipid, triglyceride decreased significantly more in the MICT group than that in the HIIT group (*P* = 0.006). VO_2peak_ increased in both the HIIT and MICT groups, but there was no significant between-group difference (*P* = 0.647).

**Conclusion:**

HIIT and MICT significantly improved blood glucose and aerobic capacity in patients with pre-diabetes. However, MICT was superior to HIIT in terms of visceral fat, lipids, and diastolic blood pressure.

**Clinical Trial Registration:**

https://www.chictr.org.cn/, identifier ChiCTR1900026905.

## Introduction

The latest national representative survey in China reveals that the estimated prevalence of pre-diabetes was 38.1% in 2018, and pre-diabetes increases the morbidity and mortality of cardiovascular and metabolic diseases (1, 2). Pre-diabetes is an abnormal metabolic state between normoglycemia and diabetes mellitus, characterized by impaired glucose tolerance and fasting glucose (3). Unhealthy lifestyles such as physical inactivity, abnormal body composition, and sedentary lifestyle play a key role in the development and progression of pre-diabetes (4). Therefore, strategies based on lifestyle interventions such as weight loss, regular exercise, and reduction of sedentary behavior have been widely recommended for the management of patients with pre-diabetes, and intensive lifestyle interventions have been shown to have a significant effect on preventing or delaying the onset and progression of type 2 diabetes mellitus (T2DM) (5–7).

Exercise as the most common lifestyle intervention has a broad spectrum of cardiovascular and metabolic protective benefits and produces clinical benefits in pre-diabetic populations by mediating various endogenous mechanisms (8, 9). Moderate-intensity continuous training (MICT) is the most common mode of exercise in people with diabetes and pre-diabetes, and guidelines recommend that this population performs a minimum of 150 min of MICT per week for health benefits (8). However, MICT faced significant challenges, because some people lacked adherence and limited motivation and time to follow these guidelines (10). High-intensity interval training (HIIT) is a training method that involves alternating short bursts of high-intensity exercise with periods of rest or lower-intensity exercise (11). The advantage of HIIT over equivalent workload of MICT is that the same or more clinical benefits as MICT can be achieved in a shorter training time. In recent years, some studies have shown that HIIT and MICT could also control blood glucose in patients with T2DM, but HIIT had an advantage in time (12). There is still confusion about which type of exercise is better for patients with pre-diabetes. Therefore, the aim of this study is to compare the effects of HIIT and MICT on body composition, cardiovascular function, cardiorespiratory function, and glucolipid metabolism in patients with pre-diabetes.

## Materials and methods

### Study design

**Figure 1:**
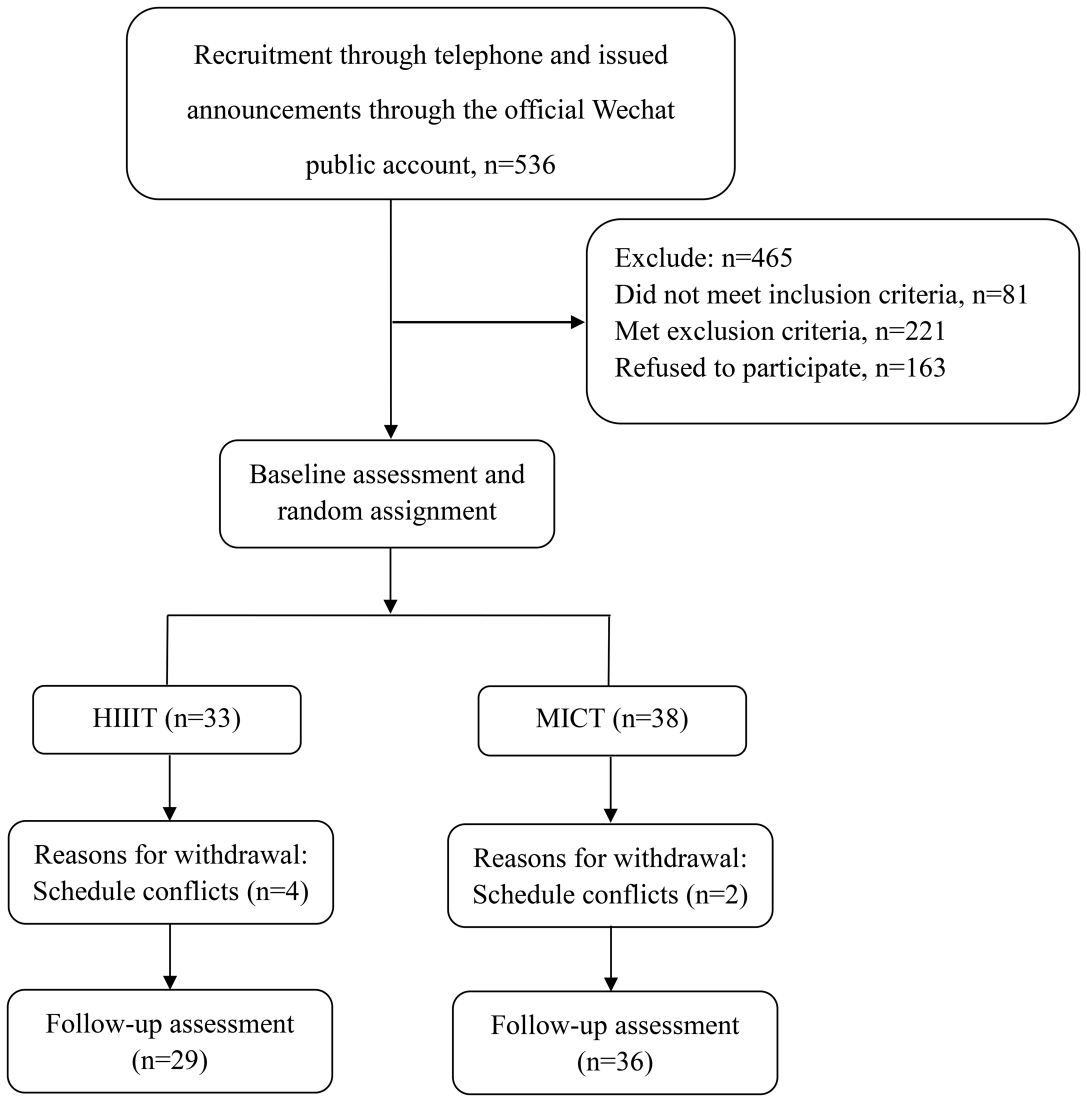
Flowchart of volunteer recruitment for the experiment.

This was an assessor-blind, two-arm, parallel randomized controlled trial. An overview of the trial procedures could be seen in **Figure 1**. The study protocol received approval from the Ethics Committee of Second Affiliated Hospital of Fujian University of Traditional Chinese Medicine (Ethics Committee Protocol Number: SPHFJP-K2019031-03) and adhered to the principles outlined in the Declaration of Helsinki. The trial was registered on the Clinical Trial Registry (ChiCTR1900026905). Before enrollment, all participants provided written informed consent, and the study’s findings were reported by the Consolidated Standards of Reporting Trials (CONSORT) guidelines.

### Research setting

The medical examination database of the Second Affiliated Hospital of Fujian University of Traditional Chinese Medicine screened the patients of pre-diabetes for telephone recruitment and issued recruitment announcements through the official WeChat public account. The experimental procedures and potential risks were explained to patients before they participated in the study. Written informed consent was obtained from all the participants. After baseline assessment, researchers, external to the project, used the computer-generated random number list with a 1:1 allocation ratio. Allocation of participation to the HIIT and MICT groups was performed by the study coordinator. In addition, health talks on dietary recommendations were conducted for all participants, which were based on the Dietary Guidelines for Chinese Residents, version 2022, for controlling overall energy intake and the balance of various dietary categories.

### Participant inclusion

Participants were considered eligible on the basis of the following criteria: 1) diagnosis of pre-diabetes; 2) 45–59 years of age; 3) no contraindications to exercise treadmill tests; and 4) not engaging in regular exercise (30-min aerobic exercise, three times a week) for at least 3 months prior to the commencement of the exercise program.

### Exclusion criteria

Participants who met any of the following criteria would be excluded from the study: 1) pregnancy or lactation; 2) history of heart disease and severe lung disease; and 3) history of malignant tumor.

### Random assignment and blinding

Following the initial baseline assessment, the participants were randomly allocated into the HIIT group and the MICT group in a 1:1 ratio. This study was conducted as an assessor-blind randomized controlled trial, with measurements taken at baseline and after 12 weeks by assessors who were unaware of the participants’ group assignments. The interventions were administered by the study staff. The randomization table was generated by the Department of Statistics at the School of Public Health, Fudan University, using Microsoft Excel software. The study staff, who remained unaware of the group allocations, collected the measurements and maintained possession of the randomization table. The group assignments for each participant were sealed in opaque envelopes and were only revealed after the completion of the baseline assessments.

### Intervention

Following the completion of informed consent and screening procedures, participants were randomized in a 1:1 ratio to either the MICT group or the HITT group. To ensure the minimal effective dose of exercise, a minimum attendance rate was set on the basis of previous research findings (13). More specifically, participants who attended fewer than 80% of the training sessions were considered dropouts. Adherence to the training program was closely monitored and consistently documented throughout the duration of the trial, enabling a thorough and ongoing analysis and assessment. Participants in both the MICT and HIIT groups engaged in cycle ergometer training with varying intensity three times a week. Exercise intensity, duration, and heart rate (HR) were recorded during exercise, and adverse events that occurred during exercise were recorded.

### HIIT group

HIIT session consisted of 5-min warm-up and 5-min cool-down (50%–60% HR_peak_) and 10 1-min intervals at high intensity (75%–90% HR_peak_) for a Borg Rating of Perceived Exertion (RPE) scale rating of 16–17 and interspersed with 10 1-min intervals at moderate exercise (50% HR_peak_) for a Borg RPE scale rating of 10–12. The total training duration was 30 min. Throughout the training sessions, the resistance of the cycle ergometer was adjusted continuously to ensure that each session was carried out at the designated HR.

### MICT group

Each MICT session comprised a 5-min warm-up and a 5-min cooldown phase (50%–60% HR_peak_), with 50 min of moderate exercise (55%–70% HR_peak_) for a Borg RPE scale rating of 13–15 in between. A HR monitor was utilized to measure the training HR. Throughout the training sessions, the resistance of the cycle ergometer was adjusted continuously to ensure that each session was carried out at the designated HR.

### Measurements

Blinded research assistants gathered measurements at baseline and following the 12-week intervention, including body composition, cardiovascular index, glycolipid metabolism, and cardiopulmonary function index. In addition, the participants would have an examination of fasting blood glucose (FBG) and 2-h glucose after 6-week training. The brachial blood pressure of participants was measured using an electronic sphygmomanometer (Omron, HBP-9021). The body composition of the patients was measured using a body fat measuring instrument (TANITA, MC-980MA) through the bioelectrical impedance analysis method. The glycolipid metabolism of the patients was measured using the automatic biochemical analyzer (Abbott, C16200). Cardiovascular indicators were examined using an ultrasonic color Doppler diagnostic instrument (PHILIPS, EPIQ7 EXP). The cardiopulmonary function indicator VO_2peak_ was measured through incremental load running on a treadmill, and the treadmill exercise protocol adopted the modified Bruce protocol.

### Statistical analysis

Data analysis was performed using IBM SPSS Statistics version 27.0 and GraphPad Prism 10.2.1, and all data of this study were expressed as mean ± standard deviation (SD) and using a two-sided 5% level of significance. The Kolmogorov–Smirnov test was used to test the normality of the distribution of the data. Participants’ characteristics were analyzed using an independent sample t-test if the data were normal, otherwise using the Wilcoxon rank sum test to assess potential differences at baseline and follow-up data between the two groups. The paired sample t-test or Wilcoxon rank sum test was used to assess (baseline and follow-up) within group according to the normal distribution of the data.

## Result

### Participants’ baseline condition

The participant flow is illustrated in **Figure 1**. A total of 71 participants met the inclusion criteria and were randomized to the HIIT and MICT groups, of which 91.55% completed the study. Approximately 12.12% of participants in HIIT and 5.26% in MICT discontinued the exercise intervention (*P* = 0.3). Participants’ characteristics for those who completed the study were presented, and the participants’ baseline conditions were not statistically different between groups in **Table 1**.

**Table 1:** Baseline participant characteristics of pre-diabetic patients in the HIIT and MICT groups.

Parameter	HIIT (n = 29)	MICT (n = 36)	*P*-value
Age	51 ± 4.42	51.69 ± 4.61	0.174
Male, n (%)	17 (52.78)	19 (65.51)	0.638
Height (cm)	165.81 ± 7.72	164.75 ± 7.26	0.230
Weight (kg)	66.76 ± 8.59	67.21 ± 7.32	0.538
BMI (kg/m^2^)	23.76 ± 1.39	24.69 ± 1.98	0.398
FBG (mmol/L)	6.32 ± 0.47	6.37 ± 0.34	0.548
HbA1c (%)	5.79 ± 0.43	5.99 ± 0.66	0.791
VO_2peak_ (ml/kg/min)	35.34 ± 5.53	31.81 ± 4.98	0.327
Blood pressure mean			
Systolic (mmHg)	119.48 ± 9.43	121.25 ± 13.5	0.243
Diastolic (mmHg)	72.83 ± 7.93	75.08 ± 10.48	0.741
Body composition			
Lean mass (kg)	49.38 ± 8.82	48.72 ± 8.48	0.428
Fat mass (kg)	16.43 ± 4.37	18.44 ± 4.61	0.633
Muscle mass (kg)	46.71 ± 8.48	46.06 ± 8.16	0.428
Body fat rate (%)	25.25 ± 6.83	27.71 ± 7.16	0.649
Visceral adipose area (kg)	2.58 ± 0.99	3.04 ± 0.9	0.746

### Comparison of body composition between groups after exercise intervention

**Figure 2:**
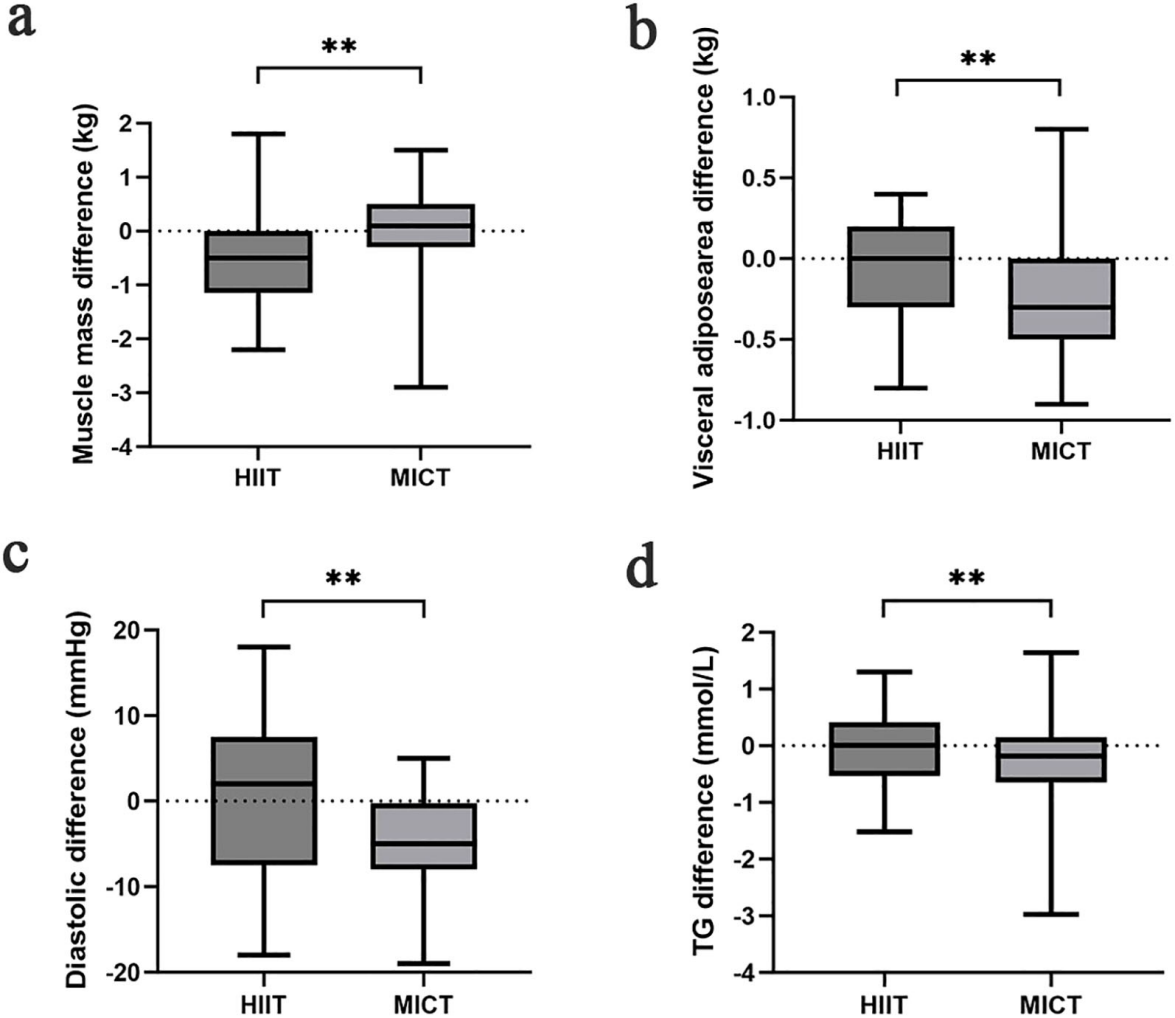
Comparison of muscle mass **(a)**, visceral adipose area **(b)**, diastolic **(c)**, TG **(d)** difference in each group after the intervention; ^*^ *p* < 0.05 and ^**^ *p* < 0.01 for statistically significant differences.

The effect of training on body composition for HIIT and MICT groups is presented in **Table 2**. The body mass index (BMI) before and after the HIIT intervention were 24.13 ± 1.52kg/m^2^ and 23.79 ± 1.47kg/m^2^, respectively. Statistical differences were observed within groups (difference = −0.35 ± 0.73 kg/m^2^, *P* = 0.016). In addition, BMI analysis revealed a statistical difference with a range of 24.79 ± 1.83kg/m^2^ before the MICT exercise intervention and a range of 24.49 ± 1.99kg/m^2–^12 weeks after the exercise intervention (*P* = 0.021). The overall means of the data in the HIIT and MICT were not statistically different (*P* = 0.787). The waist hip ratio of participants in the HIIT group was not significantly change before and after exercise. In the MICT group, the waist hip ratio of participants before and after the intervention in this group was 0.91 ± 0.03 and 0.90 ± 0.03, with a statistically significant difference between the two data (*P* = 0.01). Compared with MICT, lean mass (*P* = 0.01, Cohen’s d = 0.904) and muscle mass (*P* = 0.01, Cohen’s d = 0.857) (**Figure 2**) of participants decreased significantly in the HIIT group after 12 weeks of training, and the effect size attained Cohen’s threshold for a large effect (d ≥ 0.80). Analysis of visceral adipose area and body fat rate revealed that the participants in the MICT group had a significantly decreased in visceral adipose area, but the effect size was small (*P* = 0.043, Cohen’s d = 0.371) (**Figure 2**). The body fat rate (*P* = 0.030, Cohen’s d = 1.538) after training compared with the HIIT group, and the effect size attained Cohen’s d threshold a large effect.

**Table 2:** Change in participants’ characteristic body composition, cardiovascular index, blood glucose, blood lipid, and cardiopulmonary function index control.

Parameter	HIIT				MICT				Cohen’s d	Between-group *p*-value
	Pre	Post	d	Within-group *p-*value	Pre	Post	d	Within-group *p-*value		
Body composition										
BMI (kg/m^2^)	24.13 ± 1.52	23.79 ± 1.47	−0.35 ± 0.73	0.016^**^	24.79 ± 1.83	24.49 ± 1.99	−0.30 ± 0.75	0.021^**^	0.741	0.787
Waist hip ratio	0.90 ± 0.01	0.90 ± 0.03	0.00 ± 0.02	0.332	0.91 ± 0.03	0.90 ± 0.03	−0.01 ± 0.02	0.010^**^	0.023	0.167
Lean mass (kg)	49.38 ± 8.82	48.87 ± 8.79	−0.51 ± 0.88	0.004^**^	48.72 ± 8.48	48.7 ± 8.37	−0.02 ± 0.92	0.886	0.904	0.010^††^
Fat mass (kg)	16.43 ± 4.37	16.27 ± 4.21	−0.17 ± 1.03	0.382	18.44 ± 4.61	17.69 ± 4.76	−0.75 ± 1.48	0.004^**^	1.298	0.078
Muscle mass (kg)	46.71 ± 8.48	46.22 ± 8.45	−0.49 ± 0.84	0.004^**^	46.06 ± 8.16	46.05 ± 8.07	−0.01 ± 0.87	0.924	0.857	0.010^††^
Visceral adipose area (kg)	2.58 ± 0.99	2.53 ± 0.90	−0.05 ± 0.34	0.452	3.04 ± 0.90	2.80 ± 0.90	−0.24 ± 0.39	0.001^**^	0.371	0.043^†^
Body fat rate (%)	25.25 ± 6.83	25.27 ± 6.95	0.02 ± 1.31	0.933	27.71 ± 7.16	26.88 ± 7.25	−0.83 ± 1.7	0.006^**^	1.538	0.030^†^
Cardiovascular function										
Ejection fraction (%)	65.31 ± 2.7	64.93 ± 2.84	−0.38 ± 3.95	0.517	64.29 ± 2.58	65.06 ± 2.81	0.77 ± 4.02	0.259	3.991	0.253
IVST (mm)	0.93 ± 0.88	0.95 ± 0.87	0.02 ± 0.11	0.323	0.94 ± 0.12	0.99 ± 0.12	0.05 ± 0.12	0.020^*^	11.775	0.348
LVLd (mm)	4.27 ± 0.80	4.38 ± 0.41	0.11 ± 0.91	0.517	4.39 ± 0.30	4.37 ± 0.43	−0.03 ± 0.51	0.769	0.714	0.926
Systolic (mmHg)	119.48 ± 9.43	118.21 ± 12.15	−1.28 ± 12.54	<0.001^**^	121.25 ± 13.5	117.56 ± 13.76	−3.69 ± 10.37	<0.001^**^	11.382	0.398
Diastolic (mmHg)	72.83 ± 7.93	73.03 ± 9.57	0.21 ± 9.81	0.074	75.08 ± 10.48	69.81 ± 10.38	−5.28 ± 5.93	<0.001^**^	7.892	0.011^†^
Glycolipid metabolism										
FBG (mmol/L)	6.32 ± 0.47	5.85 ± 0.64	−0.46 ± 0.70	<0.001^**^	6.37 ± 0.34	5.97 ± 0.39	−0.39 ± 0.29	<0.001^**^	0.512	0.626
2-h glucose (mmol/L)	8.16 ± 1.94	7.29 ± 1.96	−0.87 ± 1.99	<0.001^**^	9.15 ± 2.83	7.97 ± 2.07	−1.18 ± 2.01	<0.001^**^	1.998	0.979
HbA1c (%)	5.79 ± 0.43	5.84 ± 0.39	0.05 ± 0.23	<0.001^**^	5.99 ± 0.66	5.95 ± 0.36	−0.04 ± 0.43	<0.001^**^	0.355	0.715
TG (mmol/L)	1.69 ± 1.04	1.43 ± 0.62	−0.27 ± 0.99	0.159	2.33 ± 1.86	1.47 ± 0.86	−0.86 ± 1.43	0.001^**^	1.252	0.006^††^
TC (mmol/L)	5.02 ± 0.9	4.97 ± 0.76	−0.04 ± 0.64	0.726	5.41 ± 0.99	5.15 ± 0.83	−0.27 ± 0.89	0.079	0.788	0.291
LDL (mmol/L)	2.94 ± 0.92	3.06 ± 0.68	0.12 ± 0.73	0.393	3.12 ± 0.76	3.15 ± 0.72	0.03 ± 0.64	0.798	0.681	0.895
HDL (mmol/L)	−0.09 ± 0.6	1.26 ± 0.58	−0.09 ± 0.60	0.428	1.26 ± 0.28	1.26 ± 0.22	0.00 ± 0.14	0.991	0.415	0.602
Cardiopulmonary function index										
VO_2peak_ (ml/kg/min)	35.34 ± 5.53	37.47 ± 7.34	2.13 ± 4.94	<0.001^**^	31.81 ± 4.98	33.44 ± 5.90	1.63 ± 3.40	<0.001^**^	4.156	0.647

Values are mean ± SD; the paired sample t-test or Wilcoxon rank sum test was used to assess within group according to whether the normal distribution of the data; ^*^ *p* < 0.05 and ^**^ *p* < 0.01 for statistically significant differences. Participants’ characteristics were analyzed using independent sample t-test if the data were normal otherwise using the Wilcoxon rank sum test to assess potential differences between the two groups. Cohen’s d was used as the effect size measure to assess the mean differences between groups. Based on Cohen’s (1988) conventions, effect sizes Cohen’s d were interpreted as follows: small (d = 0.20), medium (d = 0.50), and large (d ≥ 0.80). ^†^ *p* < 0.05 and ^††^ *p* < 0.01 for statistically significant difference between groups. Abbreviations: LVLd, diastolic left ventricular diameter; TG, triglyceride; TC, total cholesterol; LDL, low-density lipoprotein; HDL, high-density lipoprotein.

### Comparison of cardiovascular index between groups after exercise intervention

The interventricular septum thickness (IVST) of participants in the MICT group was thicker than that before intervention (difference = 0.05 ± 0.12, *P* = 0.02), but there was no significant difference between groups (*P* = 0.348). Analysis of systolic blood pressure indicators revealed that participants in both the HIIT and MICT groups had significantly lower values after the intervention than before (*P* < 0.001). But there was no statistically significant difference in the systolic blood pressure between the two groups (*P* = 0.398). Analysis of diastolic blood pressure indices revealed statistically significant differences within the MICT group (difference = −5.28 ± 5.93, *P* < 0.001) before and after 12 weeks of exercise intervention. MICT was better than HIIT in reducing diastolic blood pressure, and the effect size was large (*P* = 0.011, Cohen’s d = 7.892) (**Figure 2**).

### Comparison of glycolipid metabolism between groups after exercise intervention

**Figure 3:**
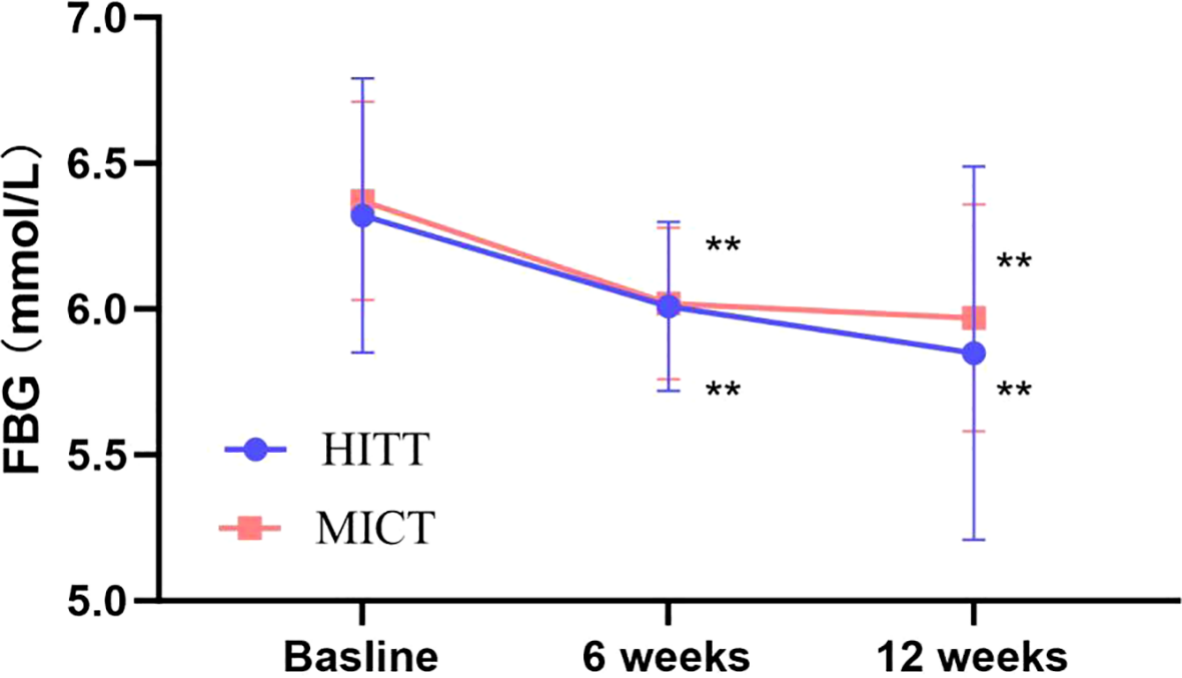
Follow-up mean FBG for the HIIT group and the MICT group. The paired sample t-test was used to evaluate the change of FBG within the HIIT and MICT groups; ^*^ *p* < 0.05 and ^**^ *p* < 0.01 for statistically significant differences in each group.

**Figure 4:**
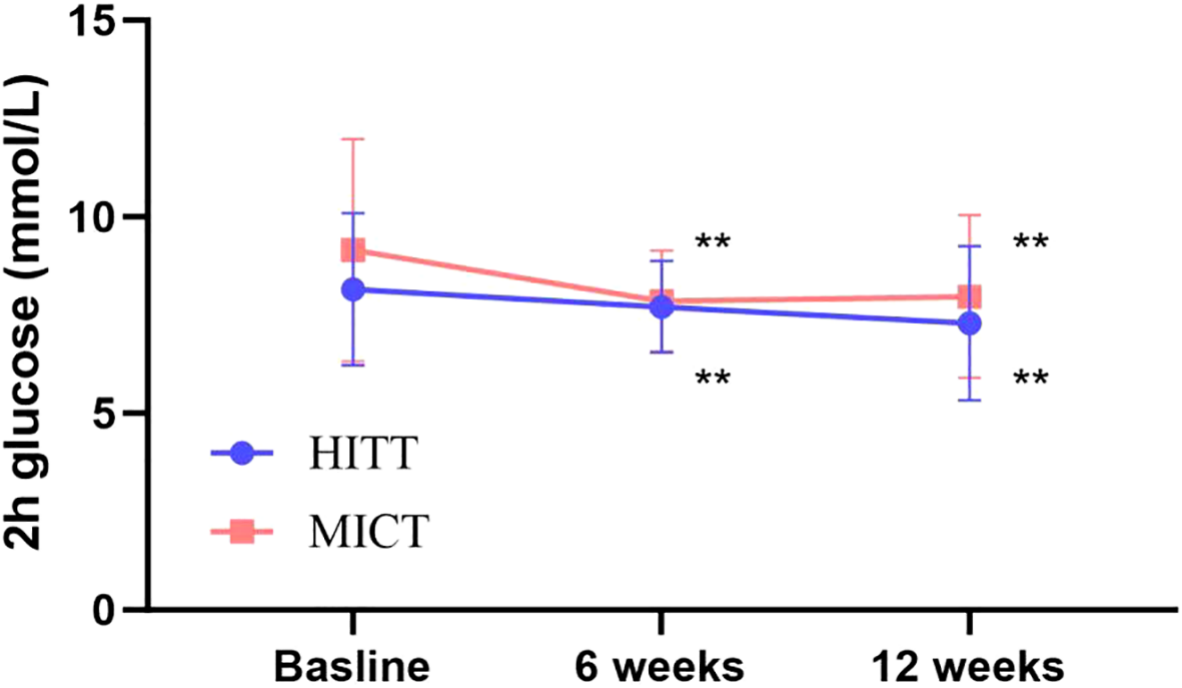
Follow-up mean 2-h glucose for the HIIT group and the MICT group. The paired sample t-test was used to evaluate the change of 2-h glucose within the HIIT and MICT groups; ^*^ *p* < 0.05 and ^**^ *p* < 0.01 for statistically significant differences in each group.

The impact of exercise interventions on patients’ blood index was presented in **Table 2**. The significant effect of FBG, 2-h glucose, and HbA1c showed an obvious descent in the HIIT and MICT groups (*P* < 0.001) before and after intervention, but no significant difference between groups was observed. In addition, FBG (**Figure 3**) and 2-h blood glucose (**Figure 4**) decreased significantly compared with the baseline in the HIIT and MICT groups, after 6 weeks of exercise. Regarding the blood lipid, triglyceride (TG) decreased significantly more in the MICT group than that in the HIIT group, with a between-group significant difference (*P* = 0.006, Cohen’s d = 1.252) (**Figure 2**), and the effect size attained Cohen’s d threshold a large effect. However, MICT and HIIT had no significant effect on total cholesterol (TC), low-density lipoprotein (LDL), and high-density lipoprotein (HDL) (P > 0.05).

### Change in cardiopulmonary function index

The impact of the exercise intervention on participants’ cardiopulmonary function index is presented in **Table 2**. There was statistical significance in the increase of VO_2peak_ in both the HIIT and MICT groups, with a mean change of 2.13 ± 4.94ml/kg/min and 1.6 ± 3.40ml/kg/min, respectively, with no significant between-group difference (*P* = 0.647).

## Discussion

This is the first study to analyze the effectiveness of HIIT and MICT on body composition, cardiovascular function, glycolipid metabolism, and cardiopulmonary function in patients with pre-diabetes.

Our study found that both HIIT and MICT were effective in reducing visceral fat area, body fat percentage, and overall BMI levels in pre-diabetic patients. The reduction in body fat percentage was more pronounced in participants in the MICT group compared to those in the HIIT group, suggesting that MICT is superior to HIIT in improving body composition (14). It has been found that even small reductions in body weight can produce a powerful multiorgan insulin-sensitizing effect, which, in turn, improves overall glucose and lipid metabolism (15). Similarly, studies showed that, in patients with T2DM, there is insufficient evidence that HIIT reduces weight compared with MICT and that the role of HIIT in weight loss should not be exaggerated (12, 16). That was consistent with our findings. Study has confirmed that moderate-intensity aerobic exercise, especially when the intensity is 60%–65% of the maximum oxygen uptake, has the highest amount of fat oxidation (17). In our study, participants in MICT decreased more visceral adipose area and body fat rate. MICT sustained training may be associated with lower energy expenditure, but it leads to increased lipolysis, resulting in the release of free fatty acids and subsequent fat oxidation, and, in this type of exercise, fat becomes the main source of fuel (18). The lean mass and muscle mass of participants decreased in the HIIT and MICT groups after 12 weeks of training, especially in the HIIT group. Exercise may elevate catecholamine levels that can decrease appetite after exercise in individuals and lead to reduced intake (19). The patients with pre-diabetes may consciously reduce carbohydrate intake to control their blood glucose levels. Carbohydrate intake can promote insulin secretion, and hyperinsulinemia can stimulate myofibrillar protein synthesis. The present study has found that hyperaminoacidemia and hyperinsulinemia exert a stimulatory effect on myofibrillar protein synthesis (20). Studies have found that carbohydrate intake inducing a moderate secretion of insulin resulted in increased myofibrillar protein synthesis rate similar to that gained after protein intake (21). That might lead to the lean mass and muscle mass of participants decreased after 12 weeks of training. In addition, the greater reduction in lean mass and muscle mass in HIIT versus MICT could potentially result from inadequate dietary control and bias due to limited sample size. In order to maintain muscle mass, we should ensure adequate protein and high-quality carbohydrate intake while exercising and can add some strength training to promote muscle synthesis.

Pre-diabetes–induced vascular disease is an important factor leading to increased risk of cardiovascular disease and all-cause mortality, and disorders of glucose and lipid metabolism play an important role in pre-diabetes–induced vascular pathology (22). Our study confirmed that both HIIT and MICT could significantly reduce FBG, 2-h glucose, and HbA1c levels of pre-diabetic patients, suggesting the potential of both types of exercise in improving glucose metabolism in this population. Exercise improves glucose metabolism through a variety of biological mechanisms. Skeletal muscle, as the most direct organ to respond to exercise and the most important organ to regulate glucose metabolism, can improve insulin sensitivity and control blood sugar by regulating muscle glycogen homeostasis (23). Our study found no difference between HIIT and MICT in improving pre-diabetic glucose metabolism, which is consistent with other studies comparing HIIT and MICT for improving glucose metabolism (24). We demonstrated the clinical benefit of HIIT and MICT in improving glucose metabolism. However, this study found that HIIT had no significant effect on circulating lipoprotein in pre-diabetic patients, and MICT only reduced TG levels. Non-response of circulating lipoproteins to HIIT and partial response of MICT have been confirmed by some preclinical studies (25). Differential results in glucose and lipid metabolism may be related to the insufficient total exercise dose in this study; in addition, this suggests that improving lipid metabolism may need to be combined with other lifestyle interventions. Therefore, based on the results of glucose and lipid metabolism, MICT may be a superior exercise option to HIIT.

Abnormal regulation of blood pressure homeostasis is considered a potential risk factor in the pre-diabetic population, and lower blood pressure levels are strongly associated with a reduced risk of cardiovascular and cerebrovascular morbidity and all-cause mortality (26–28). Relevant studies have confirmed that pre-diabetes induces vascular dysfunction through pathological mechanisms such as oxidative stress, pro-inflammatory factors, and expression of cell adhesion molecules, which, in turn, disrupts blood pressure homeostasis (29–31). In our study, we found that 12 weeks of MICT and HIIT interventions reduced systolic and diastolic blood pressure levels in the pre-diabetic population, a result confirmed by other studies (32). In addition, we further found that participants in the MICT group had a greater decrease in diastolic blood pressure. In addition, the repetitive shear stress included by exercise could enable potassium channels of the endothelial cells to become more sensitive to shear stress. Moreover, shear stress activates potassium channels that could, in turn, facilitate calcium influx into the endothelial cells (33). The increase of intracellular calcium promotes nitric oxide production for vasodilation (34). Our study found that in the MICT group, the IVST thickened after 12 weeks of training compared to before. The study of Wundersitz et al. also observed an increase in IVST thickness in athletes after cycling endurance exercise, which is consistent with our findings (35). That may be attributed to long-term endurance aerobic exercise, where the heart needs to sustain a high cardiac output for extended periods, and the increased ventricular filling volume and greater cardiac volume load can lead to adaptive changes in both the left and right ventricles (36). The absence of significant IVST changes in the HIIT group before and after intervention may be attributed to the relatively short 12-week training duration and the limited sample size, which could have introduced some bias in the results.

It is well-known that VO_2peak_ is an important indicator for evaluating cardiorespiratory function and a major predictor of cardiovascular mortality and all-cause mortality (37). Low VO_2peak_ levels are associated with abnormal glucose metabolism, which is an important risk factor for pre-diabetes and new-onset diabetes (38). We found that both HIIT and MICT could significantly improve VO_2peak_ levels, and these results have important clinical implications, which suggest the potential value of HIIT and MICT in improving cardiorespiratory function and reducing cardiovascular mortality in pre-diabetic populations. Some studies have confirmed that HIIT may be superior to MICT in improving VO_2peak_ of the pre-diabetic population, which may result from the more extensive oxygen consumption mediated by HIIT (39). Our study found that there was no difference between HIIT and MICT in improving VO_2peak_, which may be related to the dose of our research exercise model.

### Limitations

Our study has certain limitations that should be acknowledged. Firstly, although we conducted dietary education for patients with pre-diabetes, we did not strictly monitor and record the diet of patients. The patient’s diet may impact on their health- related indicators. Secondly, we did not strictly control for energy expenditure equivalence between the MICT and HIIT protocols, which could influence the observed outcomes. We will use the HR-based energy expenditure estimation to ensure better comparability between groups in future research. Thirdly, the duration of our exercise intervention was only 12 weeks, and there was no long-term follow-up on whether either exercise regimen reversed pre-diabetes or delayed the onset of diabetes.

## Conclusions

HIIT and MICT significantly improved blood glucose and aerobic capacity in patients with pre-diabetes. However, MICT was superior to HIIT in terms of visceral fat, lipids, and diastolic blood pressure.

## Data Availability

The raw data supporting the conclusions of this article will be made available by the authors, without undue reservation.
